# Atrial fibrillation and acute myocardial infarction: a Two-Way relationship

**DOI:** 10.3389/fcvm.2026.1699897

**Published:** 2026-02-27

**Authors:** Arianna Pannunzio, Flavio Mastroianni, Laura Gatto, Pasquale Pignatelli, Francesco Prati, Daniele Pastori, Danilo Menichelli, Flavio Giuseppe Biccirè

**Affiliations:** 1 Department of General Surgery, Surgical Specialty and Anesthesiology Paride Stefanini, Sapienza University of Rome, Rome, Italy; 2 Cardiovascular Sciences Department, San Giovanni Addolorata Hospital, Rome, Italy; 3 Centro per la Lotta Contro L’Infarto—CLI Foundation, Rome, Italy; 4 Department of Medical and Cardiovascular Sciences, Sapienza University of Rome, Rome, Italy; 5 UniCamillus—Saint Camillus International University of Health Sciences, Rome, Italy; 6 IRCCS Neuromed, Località Camerelle, Pozzilli, Italy

**Keywords:** atherosclerosis, atrial fibrillation, cardiovascular events, late atrial fibrillation, myocardial infarction

## Abstract

Atrial fibrillation (AF) and coronary artery disease (CAD) are among the most frequent cardiovascular diseases and leading causes of morbidity/mortality worldwide. The concomitant presence of AF and CAD is relatively common, as the association is supported not only by shared atherosclerotic risk factors, but also by a pathophysiological link. Patients with a history of AF have been described as at increased risk of CAD, in particular acute myocardial infarction (AMI), through several mechanisms, such as increased oxidative stress, systemic inflammation, increased platelet aggregation. On the other hand, up to 10% of patients with AMI are at risk of developing new-onset atrial fibrillation (NOAF). In the past, any type of NOAF during AMI was considered identical and equally associated with a worse outcome. More recently, increasing evidence supports the pathophysiological and nosological difference between early NOAF (occurring within the first 24 h after the index event and associated with atrial ischaemia, oxidative stress and a better outcome) and late NOAF (occurring after 24 h and correlated with increased left atrial pressures, deterioration of haemodynamic status, elevated left ventricular filling pressures and a worse outcome). In this review, we summarise the available evidence on the epidemiology, pathophysiology, risk stratification, and management of the complex two-way relationship between AF and CAD.

## 1 Introduction

Atrial fibrillation (AF) is the most frequent arrythmia which carries an increased risk of cardiovascular events (CVEs), disability, hospitalisations and mortality (1). Patients suffering from AF are indeed at substantially increased risk of thromboembolic stroke (five-fold increase) and CVEs (2, 3), including acute coronary syndromes (ACS) (2, 4, 5) and heart failure (HF) (6). On the other hand, 10% of patients admitted with acute myocardial infarction (AMI) develop new-onset AF (NOAF) during the hospitalisation (7). This two-way relationship represents a challenge in clinical practice due to the high morbidity and mortality related to the coexistence of these disorders. Recently, several integrated approaches were proposed to improve clinical outcome and prognosis, using a comprehensive approach (8, 9). In this review, we summarised the risk of new-onset myocardial infarction (MI) in patients with AF and the risk of NOAF in patients with AMI, exploring the two-way relationship between these common and high mortality rate-related disorders.

## 2 AF and risk of AMI

### 2.1 Epidemiology

Current evidence suggests that patients with AF are at increased risk of AMI, with an incidence ranging from 0.5% to 4% per year (10). This risk of AMI in AF patients is substantially increased in case of patients with stable coronary artery disease (11.5%/year), vascular disease (4.47%/year), HF (2.9%/year), and in those undergoing coronary artery interventions (6.3%/year) (11). Lower annual rates have been described in AF patients from Eastern countries (0.2%–0.3%/year), and in those enrolled in clinical trials (from 0.4% to 1.3%/year) (11). The most common manifestation of ACS in patients with a history of AF is non-ST-segment elevation myocardial infarction (non-STEMI)/unstable angina (60.3%–69.6%) (12, 13), followed by STEMI (19.6%–22.3%) (12, 13). The occurrence of CVEs in AF patients underlines the burden of atherosclerotic disease in this population and suggests that AF patients are exposed to an increased cardiovascular risk, and not only to the risk of thromboembolic events.

As mentioned, this risk is conferred by the presence of multiple shared atherosclerotic risk factors. A summary of comorbidities’ prevalence in AF and AMI patients **is listed in** Table 1.

**Table 1:** Common atherosclerotic risk factors and comorbidities between AF and AMI.

Risk factor	Prevalence in AF [ref. (1, 14, 73, 75–80)]	Prevalence in AMI [ref. (81–86)]
Diabetes (%)	19.6–31.0	16.3–37.7
Arterial hypertension (%)	59.8–88.0	44.3–69.0
Dyslipidaemia (%)	28.8–54.6	40.3–51.9
COPD (%)	4.9–13.7	7.0–30.0
Smoking (%)	9.5–24.4	24.1–30.7
Obesity (%)	28.2–35.4	24.1–29.4
Chronic kidney disease (%)	19.0–46.9	2.0–2.3
Alcohol (%)	2.2–5.4	7.2–12.5

AMI, acute myocardial infarction; COPD, chronic obstructive pulmonary disease.

Age and male sex represented the most common unmodifiable risk factors for AF and AMI. Indeed, the risks of developing AF and AMI progressively raise by aging (14, 15) and in male patients (16).

Patients with AF are usually affected by several atherosclerotic risk factors such as arterial hypertension, diabetes, overweight/obesity, and dyslipidaemia, that represent the so-called metabolic syndrome (17) associated with an increased risk of cardiovascular complications, such as ischemic stroke and coronary artery disease (CAD) (11, 18–20).

Several modifiable risk factors are also associated with both AF and AMI. Unhealthy lifestyle behaviours such as smoking and alcohol abuse are well-known risk factors for AMI and AF (16). In particular, smoking seems to increase the risk of AMI by 2.5 fold and AF by 1.3 fold (21), and its cessation has been associated with a reduction of both cardiac diseases incidence (16).

Independently by concomitant diseases and clinical risk factors, environmental factors could represent a common risk factor for both disorders (16). Firstly, a lower socio-economic status, as well as lower educational status and income (22–24) have been shown to be valid predictors of AF and AMI, influencing also the survival rate after these events (22, 25).

### 2.2 Integrated approach for AF and therapeutic prospectives

The risk of CVEs in this population has prompted the integration of comprehensive approaches to identify subjects at enhanced risk of events (26). Several integrated managements were developed to improve prognosis in patients with AF: the most known and recommended are the ABC pathway and the AF-CARE (8, 9). Both suggest a complete evaluation of patients by considering comorbidities and concomitant risk factors. These approaches include an optimal quality of anticoagulation to reduce the risk of thromboembolic stroke, a rhythm or rate control strategy to control the symptoms AF related and the management of comorbidities. Among comorbidities management, appropriate therapy to reduce the AMI risk represents an important challenge in AF integrated care approach.

An adequate adherence to ABC pathway, as evaluated by several observational studies on AF patients (27, 28) was associated with a reduction of stroke/systemic embolism [odds ratio [OR] 0.55; 95% confidence interval [95%CI] 0.37–0.82], cardiovascular death (OR 0.37; 95%CI 0.23–0.58), all-cause of death (OR 0.42; 95%CI 0.31–0.56) and major bleeding (OR: 0.69; 95% CI: 0.51–0.94) compared to AF patients with poor adherence. **On the other hand, the AF-CARE approach had similar evaluation points than ABC pathway. In addition, the “E” point (evaluation and dynamic reassessment) that is peculiar of AF-CARE, may be a strength of this score allowing a dynamic and continuous assessment of thromboembolic and haemorrhagic risk of patients with AF. However, there were few evidence and studies about this new score compared to ABC pathway and further studies are needed.**

## 3 NOAF in patients with AMI

### 3.1 Pathophysiological mechanisms and chronological aspects of NOAF

Patients with acute cardiac events—especially AMI—are at increased risk of developing NOAF compared with the general population. Over the past few years, accumulating evidence has substantiated this concept and showed that one or more episodes of newly detected AF are a relatively common complication of MI (29–31). In modern registries, the incidence of NOAF in patients with AMI varies between 2.3% and 10% (32–34). The risk of NOAF is increased by 60%–77% in patients with AMI and the presence of an unrecognized AMI represents an independent predictor of AF regardless of traditional cardiovascular risk factors (35). Several studies have demonstrated the association between AF episodes complicating AMI and increased risks of adverse events, including higher rates of death, reinfarction and ischaemic stroke (31, 32, 36, 37).

**Figure 1:**
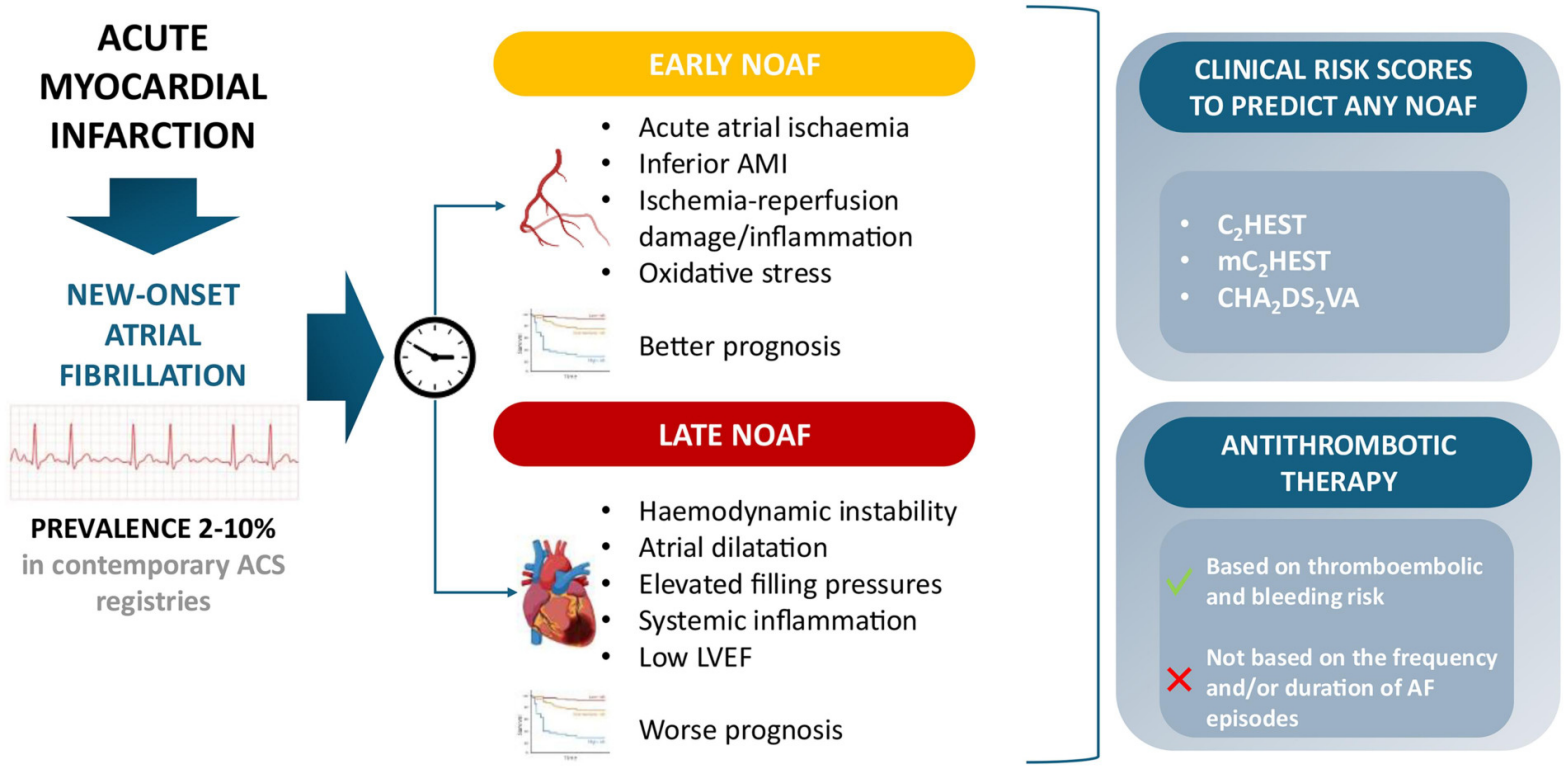
Prevalence, phenotypes, clinical risk predicting scores and antithrombotic therapy in patients with acute myocardial infarction with or at risk of new-onset atrial fibrillation.

Most studies focused on the prevalence and clinical characteristics of any NOAF episode during MI. However, two distinct phenotypes of NOAF are increasingly recognised: AF episodes occurring within the first 24 h after the acute event (early NOAF) and AF episodes occurring after 24 h from the acute event (late NOAF) (38). These two types of NOAF have been associated with different pathophysiological mechanisms and prognostic values (Figure 1) (38–42).

From a pathological point of view, the distinction between early and late NOAF appears substantial. Previous literature reported that general mechanisms underlying NOAF include diastolic and systolic left ventricular dysfunction, haemodynamic impairment, mitral valve regurgitation, right ventricular infarction, systemic inflammation, hypokalaemia and acute hypoxia (43). However, recent studies showed distinct pathophysiological pathways underpinning early vs. late NOAF. Early NOAF appears to be more related to acute atrial ischaemia, ischemia-reperfusion damage and oxidative stress following acute occlusion of coronary vessels supplying blood to atrial myocytes, sinoatrial node, atrioventricular node or other electrical signal pathways (44). Accordingly, the following parameters have been reported to be associated with shorter and earlier episodes of NOAF: younger age, more frequent inferior MI, thrombolysis, higher left ventricular ejection fraction (LVEF), less frequent HF, and smaller left atrial size (38, 40). Furthermore, unlike chronic settings where AF has been associated with less frequent multivessel disease and less frequent involvement of the right coronary artery, the presence of coronary artery disease in the right coronary artery or left circumflex (when dominant) have been described as predictors of NOAF in AMI context, especially in the initial phase (12, 40, 45). Among the transient **electrophysiologic** phenomena correlated with NOAF there is also complete atrioventricular block, usually occurring in right coronary artery-related AMI which commonly supplies blood to the atrium and the atrio-ventricular node (46). A recent study involving 155 consecutive STEMI patients undergoing primary percutaneous coronary intervention (PCI) (<12 h) **documents a** significant association between acute atrial ischemia and early but not late NOAF. At the angiographic analysis of atrial branches, 80% of patients with early NOAF showed occlusion of at least 2 atrial branches before recanalisation of the culprit vessel (20% involvement of the ramus ostia cavae superioris, 80% of the atrio-ventricular node artery, 80% of the right intermediate atrial artery, and 25% of the left intermediate atrial artery), compared with 20% or 25% of patients with sinus rhythm or late occurrence of NOAF (40).

In sharp contrast, late NOAF has been described to characterize patients with haemodynamic instability, atrial dilatation, elevated filling pressures, low left ventricular ejection fraction and cardiogenic shock (38, 40, 47). Episodes of AF occurring in unstable patients can significantly accelerate acute haemodynamic instability because of the irregular ventricular filling, loss of atrial contribution to cardiac output, and increase in oxygen demand. These factors are all strictly implicated in patient status deterioration; thus, it is not surprising that late NOAF is more associated with a worse prognosis than early NOAF.

**Despite the apparent pathophysiological differences between early and late NOAF, most available studies distinguishing these entities have primarily focused on clinical outcomes rather than on dedicated mechanistic investigations. As a result, the biological substrates underlying early vs. late NOAF remain largely inferred from clinical correlates and indirect markers, highlighting the need for future studies specifically designed to explore the mechanistic continuum linking timing of AF onset and atrial pathology after AMI.**

### 3.2 Clinical risk scores to predict NOAF

To date, the identification of patients at risk of NOAF during AMI remains challenging. Several laboratory markers, including NT-pro-brain natriuretic peptide and high-sensitivity C-reactive protein, clinical risk factors (ie. age, and sex, Killip class) and imaging variables have been associated with an increased risk of NOAF, **with** mixed results. Over the years, different clinical risk scores have also been investigated to predict the occurrence of NOAF, as they are practical and potentially more comprehensive than single markers. In a prospective study including 696 patients with STEMI, CHADS_2_ and CHA_2_DS_2_-VASc risk scores, using a cutoff point of 1.5, have been described to independently predict the development of NOAF (48). However, CHA_2_DS_2_VASc has been developed to determine the thromboembolic risk of patients with AF, and not its onset. Conversely, a new simple scoring system, namely C_2_HEST score (coronary artery disease or chronic obstructive pulmonary disease [1 point each], hypertension [1 point], elderly [age ≥75 years, 2 points], systolic HF [2 points], thyroid disease [1 point]) has recently been developed to identify the risk of overt or subclinical AF in the general population (49, 50)*.* Subsequently, a modified version of it has been proposed, including age ≥65 years as an additional variable (1 point) to the original model (51). The predictive value of these scores in the context of AMI has recently been tested. In a study on 555 patients with STEMI, C_2_HEST and mC_2_HEST scores significantly predicted the occurrence of in-hospital NOAF, showing both good accuracy (AUC 0.7 and 0.69, respectively) (52). These results were subsequently confirmed in a meta-analysis with a sample of >11 million patients, where the C_2_HEST score was robustly predictive of NOAF onset in both general population and acute settings (53).

### 3.3 Strategies to reduce the burden of NOAF

The improvement in AMI management (early reperfusion therapies, intensive care units and primary percutaneous coronary intervention) has significantly decreased the rates of NOAF AMI-related over the years (54, 55). At this regard, the use of some cardiovascular pharmaceutics has been associated with a lower incidence of NOAF. Beyond their robust benefits on plaque regression and stabilization in patients with MI (56), statins have pleiotropic effects that include dampening of NADPH oxidase activity and proinflammatory pathways, both reportedly implicated in NOAF onset. Accordingly, statin use has been also associated with lower incidence of NOAF (57, 58), yet definitive evidence at this regard is lacking. A recent metanalysis involving 48,583 participants explored the role of glucagon-like peptide-1 receptor agonist (GLP-1 RAs) in preventing NOAF, showing that treatment with semaglutide can significantly reduce the incidence of NOAF **by** about 20%–50% (59).

A recent metanalysis also showed that SGLT2i prevented AF in randomized clinical trial including patients with HFrEF (risk ratio 0.62 95%CI 0.44–0.87) (60).

Lastly, high adherence to the Mediterranean diet, rich in antioxidant-rich components, in free AF patients has been associated with sinus rhythm maintenance and with spontaneous cardioversion in those with AF, potentially suggesting an important role **of diet** in patients at risk of NOAF (61, 62).

### 3.4 Antithrombotic therapy

Beyond haemodynamic instability in the acute phase post-AMI, the main concerns regarding NOAF include short- and long-term thromboembolic risk. Past observational studies have shown that patients with AMI and AF often receive suboptimal anti-thrombotic treatment (63). To date, there is no robust evidence that pharmacological strategies can prevent AF relapses after AMI. Additionally, it is now largely acknowledged that the need for anticoagulation should be based on a thorough risk assessment, considering both thromboembolic and bleeding risks, more than on the number and duration of AF episodes (9). In AMI context, bleeding considerations are especially needed, as patients usually require dual antiplatelet therapies for both ACS secondary prevention and PCI. The 2023 European Society of Cardiology (ESC) guidelines on ACS reported a recommendation with Class IIa level of evidence C for long-term oral anticoagulation of NOAF in ACS based on the CHA_2_DS_2_-VASc score and after considering bleeding risks and concomitant antiplatelet therapy (64). More recently, the 2024 ESC consensus document on AF management recommended early cessation (≤1 week) of aspirin and continuation of an oral anticoagulant (preferably DOAC) with a P_2_Y_12_ inhibitor (preferable clopidogrel) for up to 12 months in presence of high bleeding risk and low thrombotic risk (9). Conversely, triple therapy with aspirin, clopidogrel and oral anticoagulation for longer than 1 week (typically at least 1 month) after ACS was recommended (Class IIa level of evidence C) in presence of an ischaemic risk that exceeds the bleeding risk (9).

The use of direct oral anticoagulants (DOAC) seems to be associated with a reduction of CVEs, particularly AMI, as compared to vitamin K antagonists (VKA). However, no specific clinical trials have been performed and no strong evidence at this regard is available (65). If patients are treated with VKA, an accurate control of anticoagulation quality, as reflected by a high time in the therapeutic range, may help in reducing CVEs (66). Importantly, a residual cardiovascular risk may persist despite optimal anticoagulation (19, 67), the concomitant use of antiplatelet drugs in AF patients does not seem to further reduce CVEs, and it is associated with higher rate of bleeding events (68–72).

Finally, despite the addition of renin-angiotensin inhibitors (73) and statins (74) has been associated with CVEs reduction, their use is suboptimal in AF patients. Statin treatment has been reported to be under prescribed in AF patients (74), along with the lack of AF-specific LDL cholesterol target, warranting further *ad hoc* strategies to improve patient management and reduce the atherosclerotic burden.

## 4 Conclusions

In recent years, substantial evidence has revealed **an important two-way** relationship between AF and AMI. In patients with history of AF, the risk of concomitant coronary events is substantially increased by shared risk factors and intrinsic mechanisms such as oxidative stress, platelet activation, endothelial dysfunction and direct thromboembolism. On the other hand, the prognosis and management of patients admitted with AMI are often complicated by new-onset (or newly detected) AF episodes, distinguished into early—more ischemia-related—or late—more haemodynamic status related—episodes. Specific diagnostic and preventive strategies are needed to improve risk stratification and management of patients with or at risk of both conditions.
